# Early decompression promotes motor recovery after cervical spinal cord injury in rats with chronic cervical spinal cord compression

**DOI:** 10.1038/s41598-022-14723-8

**Published:** 2022-08-24

**Authors:** Sho Okimatsu, Takeo Furuya, Masataka Miura, Yuki Shiratani, Atsushi Yunde, Takaki Inoue, Satoshi Maki, Seiji Ohtori

**Affiliations:** Department of Orthopaedic Surgery, Chiba University Graduate School of Medicine, Chiba, Japan

**Keywords:** Trauma, Experimental models of disease

## Abstract

The number of elderly patients with spinal cord injury without radiographic abnormalities (SCIWORA) has been increasing in recent years and common of most cervical spinal cord injuries. Basic research has shown the effectiveness of early decompression after spinal cord injury on the spinal cord without stenosis; no studies have reported the efficacy of decompression in models with spinal cord compressive lesions. The purpose of this study was to evaluate the effects of decompression surgery after acute spinal cord injury in rats with chronic spinal cord compressive lesions, mimicking SCIWORA. A water-absorbent polymer sheet (Aquaprene DX, Sanyo Chemical Industries) was inserted dorsally into the 4–5th cervical sublaminar space in 8-week-old Sprague Dawley rats to create a rat model with a chronic spinal compressive lesion. At the age of 16 weeks, 30 mildly myelopathic or asymptomatic rats with a Basso, Beattie, and Bresnahan score (BBB score) of 19 or higher were subjected to spinal cord compression injuries. The rats were divided into three groups: an immediate decompression group (decompress immediately after injury), a sub-acute decompression group (decompress 1 week after injury), and a non-decompression group. Behavioral and histological evaluations were performed 4 weeks after the injury. At 20 weeks of age, the BBB score and FLS (Forelimb Locomotor Scale) of both the immediate and the sub-acute decompression groups were significantly higher than those of the non-decompression group. There was no significant difference between the immediate decompression group and the sub-acute decompression group. TUNEL (transferase-mediated dUTP nick end labeling) staining showed significantly fewer positive cells in both decompression groups compared to the non-decompression group. LFB (Luxol fast blue) staining showed significantly more demyelination, and GAP-43 (growth associated protein-43) staining tended to show fewer positive cells in the non-decompression group. Decompression surgery in the acute or sub-acute phase of injury is effective after mild spinal cord injury in rats with chronic compressive lesions. There was no significant difference between the immediate decompression and sub-acute decompression groups.

## Introduction

Aging of the population has resulted in more elderly patients with cervical spinal cord injury (SCI) without bone and disc injury^1^. SCIWORA, a SCI without radiographic abnormality, was first proposed by Pang et al. who used it to define clinical symptoms of traumatic myelopathy with radiographic or computed tomographic features of spine fracture or instability and its pathogenesis has been receiving much attention^2^. Recent studies have suggested that relatively slight external force can cause SCIWORA in elderly patients who have spinal canal stenosis associated with cervical spondylotic changes, thickening of the yellow ligament, and ossification of posterior longitudinal ligament (OPLL)^1^. Chikuda et al. reported that in a randomized control trial of 72 patients with SCIWORA there was no difference in motor score after 1 year between patients with decompression within 24 h after SCI and decompression 2 weeks after injury^3^. In the present study, we used rats with cervical cord stenosis lesions created using a water-absorbent sheet as in previous reports^4–7^. The purpose of this study was to reveal the therapeutic effect behaviorally and histologically of decompression surgery after acute cervical SCI in rats with chronic stenosis, mimicking SCIWORA.

## Materials and methods

All methods below were carried out in accordance with ARRIVE guidelines and relevant regulations.

### Surgical procedure

#### Sheet insertion: asymptomatic spinal canal stenosis model

**Figure 1:**
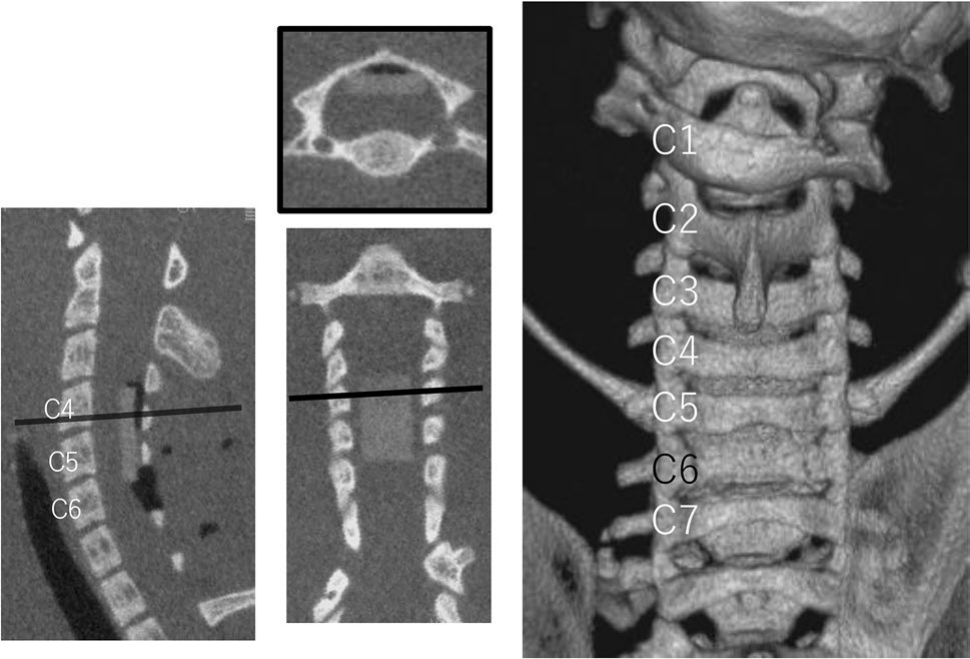
A CT taken just after the sheet insertion surgery. A C6 laminectomy was performed and a sheet was inserted on the dorsal spinal cord at the C4–C5 level.

Thirty adult female Sprague-Dawley rats (8 weeks, 180–200 g; Japan SLC, Inc., Hamamatsu, Japan) were provided with sufficient food and water in individual cages. Rats were anesthetized with sevoflurane and fixed in the prone position. An incision was made in the midline behind the neck. The C4 to C7 laminae were exposed, and the C6 laminae were removed. A 0.3 mm-thick sheet of expandable water-absorbing polyurethane elastomer Aquaprene Dx (Sanyo Chemical Industries, Kyoto, Japan) was inserted under the C4–C5 laminae using a surgical microscope. This polymer expands in volume by 200% after insertion^4,5^. A computed tomography (CT) scan was taken immediately to confirm that stenosis was created after surgery (Fig. 1).

#### Cervical SCI: contusion model

Eight weeks after the sheet insertion procedure, mild myelopathy or asymptomatic rats with a Basso, Beattie, and Bresnahan (BBB) score^8^ of 19 or higher were identified. Actually, there were no animals with a score of 18 or less that developed myelopathy. The rats were anesthetized with sevoflurane inhalation, and an incision was made in the posterior cervical vertebrae. The C4 vertebral arch was removed, confirming the sheet was just posterior to the spinal cord. A contusion injury (75 Kdyn) to the sheet at the C4–C5 level was made using the Infinite Horizon Impactor (Precision Systems and Instrumentation LLC, Notting Hill.) from 1 mm above the spinal cord. This contusion force is recognized as a mild contusion force in the rat spinal cord injury model.

### Group design

Thirty rats were divided randomly into three groups of 10 rats each:

1. Immediate decompression after SCI (immediate decompression group).
2. Decompression 1 week after SCI (sub-acute decompression group).
3. No decompression after SCI (non-decompression group).

Decompression was defined as the resection of the remaining C5 lamina and the absorbing sheet posterior to the spinal cord. To equalize the surgical invasion in each group, the immediate decompression and non-decompression groups underwent surgery under inhalation anesthesia 1 week after the SCI.

### Behavioral evaluations

Forelimb function was assessed using the Forelimb Locomotor Scale (FLS)^9^. Hindlimb motor function was evaluated using BBB scores composed of 21 different criteria for movement of the hindlimb as previously reported^8^. The scale is based on the accurate observation of coordination, joint movements, and hindlimb stepping for two min. by two observers blinded to the experimental conditions. The assessments were performed in an open field at 0, 1, 3, 7, 14, 21, 28, 35, 42, 49, and 56 days after the sheet-insertion procedure, and 0, 1, 3, 7, 14, 21, and 28 days after cervical SCI.

Mechanical withdrawal thresholds were evaluated using the Von Frey hair employing a previously reported protocol^10^. The von Frey hair was applied in ascending order of force, and the median withdrawal threshold was calculated from the values following one descending and two ascending trials.

### Histological analysis

#### Tissue preparation

Four weeks after SCI, the rats were sacrificed by transperitoneal injection with a triple mixture of 7.5 μg/100 μl of Domitor™ (medetomidine, Orion Corporation, Espoo, Finland), 40 μg/100 μl of Dormicum™ (midazolam, Maruishi Pharma, Osaka, Japan), and 50 μg/100 μl of Betolphar™ (Butorphanol Tartrate, Meiji Seika, Tokyo, Japan). The cervical spinal cord was then removed and fixed overnight in PBS (phosphate-buffered saline) with 4% formaldehyde and stored in PBS with 20% sucrose at 4 °C. Finally, the cervical spinal cord was encapsulated in an optimal cutting temperature compound (Sakura Fine Technical, Tokyo, Japan). Cryoprotected samples were frozen at − 80 °C.

#### Histological evaluation

A 2 cm long section of the cervical spinal cord centered on the C4/C5 spinal cord injury area was sectioned in horizontal (n = 5) and sagittal (n = 5) planes at a thickness of 16 μm. LFB (Luxol fast blue) staining was performed to measure the percentage of demyelinated cells in the myelin sheath of the pyramidal tract at the C7 level and to calculate the cross-sectional area of the cavity formation at the spinal cord injury. Transferase-mediated dUTP nick end labeling (TUNEL) staining was performed using an in situ Apoptosis Detection kit (Takara, Kusatsu, Japan). The number of TUNEL-positive cells in the bilateral anterior horns at the C4/5 level was counted. Axial sections were examined in triplicate, one at the center of the injury and the other at a distance of 16 μm cephalad of the center of the injury. Cell counts were averaged over the three sections. Tissue sections were co-incubated with growth-associated protein 43 (GAP-43, sc-10786, Santa Cruz Biotechnology, Inc., Dallas, US) (1:800) at 4 °C overnight followed by co-incubation with fluorescein isothiocyanate (FITC)-labeled goat anti-rabbit IgG for 1 h. at room temperature. We calculated the density of GAP43-positive cells in the immediate caudal part of the cavity formation normalized by area. All histological images were taken using an all-in-one microscope, BZ-X800, and analyzed using a BZ-X analyzer (Keyence, Osaka, Japan). The hybrid cell count function was used to calculate the number of positive cells and the percentage of demyelination to the cross-sectional area.

### Statistical and data analysis

All computations were done using the statistical package JMP (https://www.jmp.com/en_sg/software/data-analysis-software.html, version 13.2.0.; SAS Institute Inc., Cary, NC). Statistical differences in BBB score, FLS score, number of TUNEL-positive cells in the anterior horn, LFB- positive area, and the number of GAP43-positive cells were compared among the three groups using Tukey’s honest significant difference test. A p-value of < 0.05 was considered significant using two-sided tests of statistical inference.

### Ethics approval

The study was approved by the Animal Care and Use Committee of Chiba University Graduate School of Medicine (approval number 2-393).

## Results

### Contusion force

**Figure 2:**
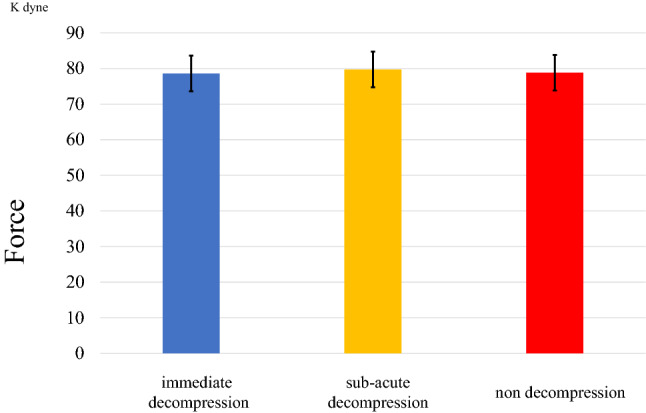
Contusion force of spinal cord injury. The contusion force of injury was not significantly different among the three groups.

The mean contusion force (± SD) creating spinal cord injury in the immediate decompression, sub-acute decompression, and non-decompression groups were 78.6 (± 2.7), 79.8 (± 2.3), and 78.8 (± 3.8) Kdyn, respectively (Fig. 2). Equal force was applied in each group, although not directly to the spinal cord but to the sheet. The original data of contusion force is shown in Supplementary Table 1.

### FLS score

**Figure 3:**
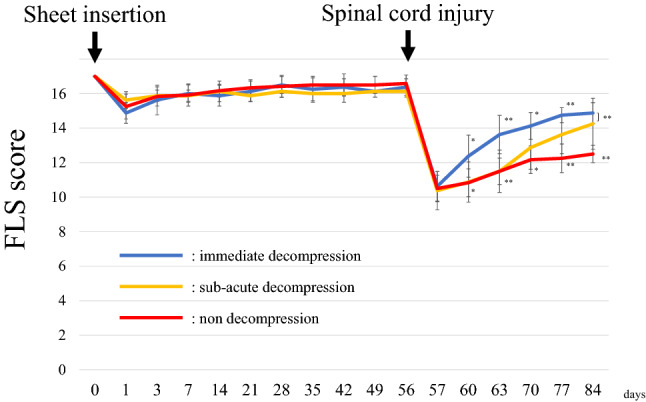
FLS score. There were no significant differences among groups before spinal cord injury. After spinal cord injury, each group improved forelimb motor function. The speed of recovery was faster in the acute decompression group than in the other groups. Four weeks after spinal cord injury, the non-decompression group had significantly lower scores than the other two groups. *FLS score* Forelimb Locomotor Scale score.

Eight weeks after sheet insertion, there was no difference between the three groups in FLS score (Fig. 3). The immediate decompression group had significantly higher scores than the non-decompression group at all time points three days after SCI. The sub-acute decompression group had significantly higher scores than the non-decompression group at 3 and 4 weeks after SCI. There was no difference in scores between the immediate decompression group and the sub-acute decompression group at the final observation. The original data of FLS score is shown in Supplementary Table 2.

### BBB score

**Figure 4:**
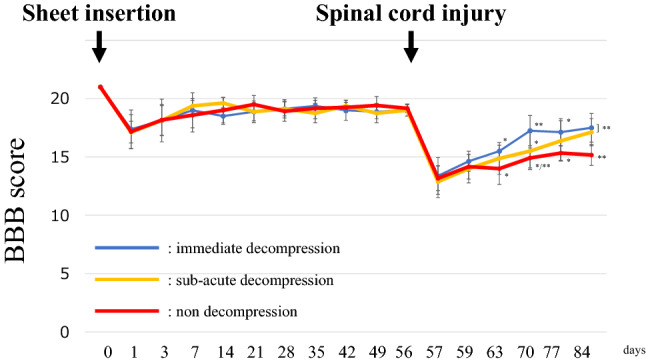
BBB score. Prior to the spinal cord injury, there were no significant differences among the groups. After spinal cord injury, each group improved hindlimb motor function. The speed of recovery was faster in the acute decompression group than in the other groups. Four weeks after SCI, the non-decompression group had a significantly lower score than the other two groups. *BBB score* Basso, Beattie and Bresnahan score.

There was no significant difference in BBB score among the three groups 8 weeks after the insertion of the sheet (Fig. 4). Eight weeks after the sheet insertion the mean ± SD BBB scores of the immediate decompression, sub-acute decompression, and non-decompression groups were 19 ± 0.5, 19 ± 0.5 and 19.2 ± 0.4, respectively. After cervical spinal cord injury, there was a difference in the recovery speed of the BBB score among three groups. The immediate decompression group had significantly higher BBB scores than the non-decompression group at all time points more than 1 week after SCI. The sub-acute decompression group had significantly higher BBB scores than the non-decompression group 2 and 4 weeks after SCI. Four weeks after injury, there was no difference between BBB scores of the immediate decompression group (17.5 ± 1.2) and that of the sub-acute decompression group (17.1 ± 1.2). The original data of BBB score is shown in Supplementary Table 3.

### TUNEL staining

**Figure 5:**
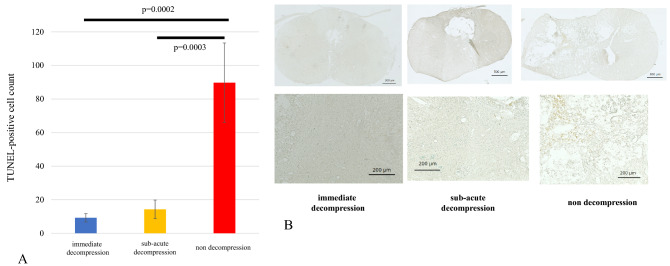
(**A**/**B**) TUNEL staining. The number of TUNEL-positive cells in the anterior horn at the level of the spinal cord injury was significantly greater in the non-decompression group. TUNEL; terminal deoxynucleotidyl transferase dUTP nick end labeling.

The number of TUNEL-positive cells in the bilateral anterior horns of the spinal cord at C4/5 in the non-decompression group was 90 ± 24, significantly higher than that of the other two groups. There were no differences in TUNEL-positive cells between the immediate decompression group (9.3 ± 2.5) and the sub-acute decompression group (13 ± 5.5) (Fig. 5A,B). The original data of TUNEL staining is shown in Supplementary Table 4.

### Demyelination

**Figure 6:**
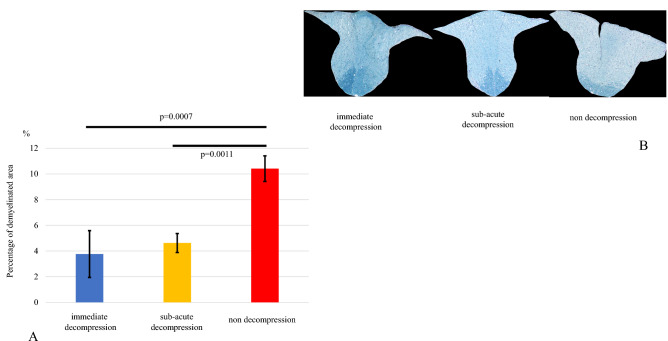
(**A**/**B**) Luxol Fast Blue staining. There was no significant difference between the immediate decompression group and the subacute decompression group in the percentage of demyelination of the posterior spinal cord at C7.

The demyelinated area divided by the total area of the posterior cord was measured to calculate the percentage of the demyelinated area of the pyramidal tract at C7. The percentage of demyelination in the non-decompression group was 10 ± 1.0%, significantly higher than that in the immediate decompression and sub-acute decompression groups, (3.8 ± 1.8% and 4.6 ± 0.74%, respectively) (Fig. 6A,B). The original data of demyelination of the pyramidal tract is shown in Supplementary Table 5.

### Sensory test: Von Frey

**Figure 7:**
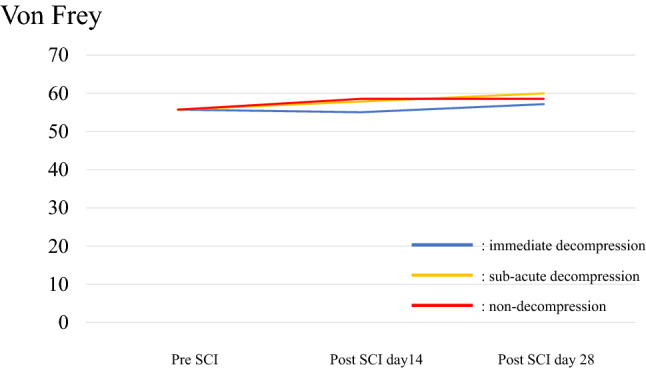
Von Frey. There were no significant differences among the groups either before or after spinal cord injury.

Throughout the entire observation period, allodynia was not significantly different among the three groups (Fig. 7). The original data of Von Frey test is shown in Supplementary Table 6.

### Cavity formation

**Figure 8:**
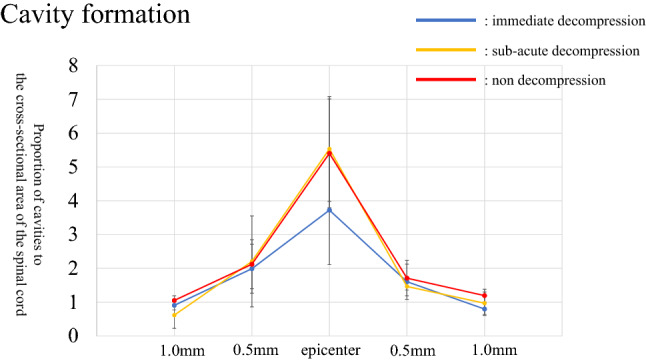
Cavity formation. In the immediate decompression group, cavity formation tended to be smaller away from the epicenter of the spinal cord injury, but there were no significant differences among groups.

There was no significant difference in the formation of cavities coincident with SCI among the three groups at the injury site, or 0.5 mm and 1 mm away from the epicenter of the damage (Fig. 8). The original data of cavity formation is shown in Supplementary Table 7.

### GAP-43

**Figure 9:**
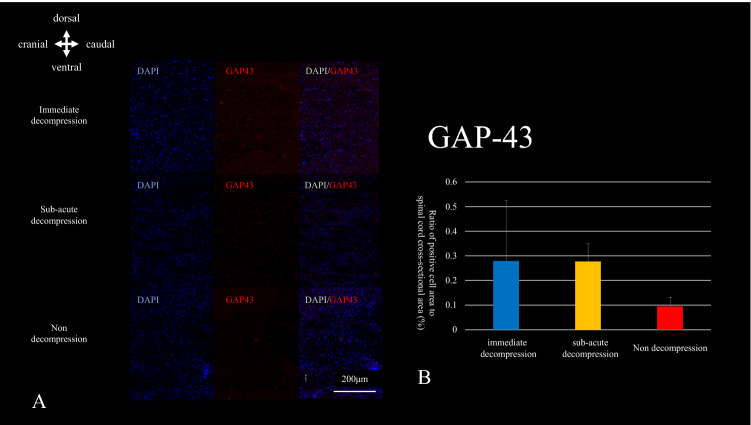
(**A**/**B**) GAP-43 staining. The number of GAP-43 positive cells located just caudal to the cavity formation tended to be lower in the non-decompressed group, but there were no significant differences among groups.

The density of GAP-43-positive cells in the area just caudal to the cavity was calculated in sagittal sections. Although there was no significant difference among the three groups, there was a trend toward fewer GAP-43-positive cells in the non-decompression group (Fig. 9A,B). The original data of GAP-43 is shown in Supplementary Table 8.

## Discussion

Decompression surgery at both acute and sub-acute phases of SCI was effective in rats with SCIWORA. Although there was no significant difference in motor function 4 weeks after injury, the speed of recovery was faster in the immediate decompression group than in the group decompressed in the subacute phase. In addition, the decompression itself was effective regardless of the timing of decompression surgery in this rat model of mild contusion injury.

The efficacy of early decompression for SCI on the normal spinal cord has been reported previously^11^. The novelty of this study is that the model of spinal cord injury used in rats with compressive lesions more closely mimics clinical practice. Moreover, there has been no prior report of a spinal cord injury model at the stenotic spinal lesion of rats. The findings in this model are similar to those reported previously in which early decompression enabled motor function to recover more quickly in humans^11^.

Decompression of the spinal cord itself was effective regardless of the timing of decompression surgery. In the current study, although the immediate decompression group recovered motor function more quickly than the sub-acute decompression group, there was no significant difference in motor or sensory function between the two decompression groups 4 weeks after cervical spinal cord injury. Both groups performed better than the non-decompression group based on BBB and FLS scores. Aarabi et al. reported that the timing of surgery does not affect the prognosis measured by the AIS (American spinal cord injury association Impairment Scale) if the spinal cord is well decompressed^12^. The finding of no difference in motor function regardless of the timing of decompression is contrary to the conclusion of Fehlings et al.^13^. In their study, about half of the patients had AIS A to B at the time of acute injury, and the post-injury pathophysiology may be different from the mild spinal cord injury model used in the present study. Batchelor et al. suggested that the duration of compression is an essential factor in determining the outcome, but only in relation to the compressive pressure^11^. A mild injury may be less time-dependent to achieve positive results from decompression surgery than a severe injury. The current model is a relatively mild to moderate rat spinal cord injury with a 75Kdyn spinal cord contusion.

In the non-decompression group, the BBB and FLS scores were significantly lower, the number of TUNEL-positive cells in the spinal cord injury area was significantly higher, and the area of demyelination in the C7 level posterior cord was significantly larger than in the decompression groups. There was a correlation between the number of TUNEL-positive cells, the degree of demyelination in the posterior cord and motor function of the limb. As there was no difference between acute decompression and sub-acute decompression, the difference in motor function between the sub-acute decompression group and the non-decompression group was possibly influenced by inflammation and cell necrosis within 1 week after the injury. It has been reported that prolonged inflammation leads to ongoing damage after spinal cord injury^14^. In a comparison of the three groups, although the immediate decompression group showed the most rapid functional improvement, there was no significant difference between the sub-acute decompression group and the immediate decompression group by 4 weeks. Clinically, in patients with mild to moderate spinal cord injury, the results suggest that decompression itself may lead to improvement in motor function, regardless of the timing of decompression surgery.

There are several limitations to this study. First, the observation period in this study was only 4 weeks after SCI, and no long-term observation was conducted. It is unclear whether the degree of improvement in motor paralysis is different more than 4 weeks after spinal cord injury. Second, the neurological deficits caused by contusion injury to the posterior aspect of the spinal cord could differ in rats and humans because the pyramidal tract of a rat is located in the posterior spinal cord whereas in humans it is located in the lateral spinal cord. Hopefully, further studies will be conducted that change the compression force and timing of decompression.

## Conclusion

Decompression surgery after SCIWORA in rats with asymptomatic canal stenosis provided better motor function than in the non-decompressed group.

## Supplementary Information

Supplementary Legends.

Supplementary Table S1.

Supplementary Table S2.

Supplementary Table S3.

Supplementary Table S4.

Supplementary Table S5.

Supplementary Table S6.

Supplementary Table S7.

Supplementary Table S8.

## Data Availability

All data generated or analyzed during this study are included in this published article and its supplementary information files.

## Abbreviations

AIS: American spinal cord injury association Impairment Scale
BBB: Basso, Beattie, and Bresnahan
CT: Computed tomography
FLS: Forelimb locomotor scale
GAP-43: Growth associated protein-43
LFB: Luxol fast blue
PBS: Phosphate-buffered saline
RNA: Ribonucleic acid
SCIWORA: Spinal cord injury without radiographic abnormality
SCI: Spinal cord injury
TUNEL: Transferase-mediated dUTP nick end labeling
