# Supplementary material for: Early decompression promotes motor recovery after cervical spinal cord injury in rats with chronic cervical spinal cord compression
Source: Sci Rep. 2022 Aug 24;12:14400. doi: 10.1038/s41598-022-14723-8 (PMC9402533; doi:10.1038/s41598-022-14723-8)
Supplement: Supplementary file 1 — Supplementary Legends. [file 41598_2022_14723_MOESM1_ESM.docx]

**Supplementary Legends**

Table. S1 BBB score

Original data of the change in BBB score from after the sheet insertion to the 4th week after spinal cord injury.

BBB score; Basso, Beattie and Bresnahan score

Table. S2 FLS score

Original data of FLS score from the time of sheet insertion to the 4th week after spinal cord injury.

FLS score; forelimb locomotor scale score

Table. S3 Contusion force

Original data of contusion force of spinal cord injury.

Table. S4 Von Frey test

Original data of the von Frey test in the hind limb after spinal cord injury.

Table. S5 TUNEL staining

Original data of TUNEL-positive cell counts at the site of spinal cord injury.

Table. S6　Luxol fast blue staining

Original data showing the percentage of demyelinated cells in the C7 posterior cord, or pyramidal tract

Table. S7 Cavity formation

Original data showing the area of cavity formation around the site of spinal cord injury.

Table. S8 GAP-43

Original data showing the number of GAP-43 positive cells in the caudal part of spinal cord injury
